# The Usefulness of the Kidney-to-Aorta Ratio in Dogs with Chronic Kidney Disease †

**DOI:** 10.3390/vetsci12010029

**Published:** 2025-01-08

**Authors:** Dario Costanza, Erica Castiello, Pierpaolo Coluccia, Camilla Sangiuliano, Maria Pia Pasolini, Micheletino Matarazzo, Giacomo Gnudi, Adelaide Greco, Leonardo Meomartino

**Affiliations:** 1 Interdepartmental Centre of Veterinary Radiology, University of Napoli “Federico II”, Via Federico Delpino 1, 80137 Napoli, Italy; 2 Department of Veterinary Medicine and Animal Production, University of Napoli “Federico II”, Via Federico Delpino 1, 80137 Napoli, Italy; 3 Department of Veterinary Science, University of Parma, Strada del Taglio 10, 43126 Parma, Italy

**Keywords:** aorta, canine, kidney injury, ratio, renal length, renal disease, ultrasonographic, ultrasound

## Abstract

The kidney length (KL) to aortic diameter (AoD) ratio (KL/AoD) has been proposed as an ultrasonographic objective method to assess renal dimensions. However, its wide range of normal values limits sensitivity. Of note, its clinical utility in detecting renal disease has not been investigated. The main aim of this study was to assess the sensitivity and specificity of KL/AoD in dogs with chronic kidney disease. Of the 227 dogs in the final sample, 185 were healthy and 42 were diseased. The obtained cut-off values for healthy dogs were similar to those previously reported. No differences were found between the KL of healthy and diseased dogs; however, using the KL/AoD ratio, diseased dogs showed a significantly smaller ratio (*p* = 0.0003), although a partial overlap between the two groups’ values was present. Considering the Youden index at KL/AoD = 6.3, the ROC curve displayed a specificity of 83.24% and a sensitivity of 41.67%, while for KL/AoD = 5.6, the specificity was 97.57% and the sensitivity was 13.10%. The results of this study provide clinical usefulness for the KL/AoD ratio method, revealing excellent specificity but poor sensitivity.

## Simple Summary

Renal dimensions are one of the parameters to evaluate during abdominal ultrasound examinations, as they can vary in the presence of diseases, such as chronic kidney disease (CKD), which is often associated with reduced renal size. In dogs, the significant variability in size among different breeds makes absolute measurements less meaningful. To address this limitation, a method where the ratio between the renal length and aortic diameter is measured has been proposed. This study assesses this method’s effectiveness in distinguishing healthy dogs from those with CKD. The results highlight the method’s high specificity: a value below the lower reference limit strongly indicates the presence of disease. However, the sensitivity is low, with many diseased dogs falling within the normal range. Therefore, this method proves particularly useful for confirming the diagnosis in dogs with clinical signs and laboratory findings consistent with CKD rather than for early screening.

## 1. Introduction

Determining renal dimensions is one of the parameters to be considered during the ultrasonographic examination of the urinary system, as it could provide useful clinical information [1,2,3,4]. Some pathologic conditions, such as acute kidney disease, compensatory hypertrophy, portosystemic shunts, pyelonephritis, amyloidosis, hydronephrosis, and neoplasia, can cause slight to severe nephromegaly [2,5]. Other conditions such as chronic kidney disease (CKD), congenital hypoplasia, and renal dysplasia can determine slight to severe reduction [2,5]. In dogs, absolute renal measurements have poor diagnostic clinical value due to the considerable variation in body weight and conformation [2,3,6]. To account for this variability, linear values of healthy kidneys, using body weight and conformations as variables, were proposed [4]. Methods where the kidney length (KL) is compared to another anatomical structure have also been proposed [7,8]. In the method by Mareschal et al., the KL is divided by the aortic luminal diameter (AoD) [7]. The proposed KL/AoD ratio method resulted in a wide range of normal cut-off values (5.5–9.1), resulting in possible overlap between healthy dogs and those with renal disease, consequently limiting the method’s clinical usefulness [2,3,9]. Furthermore, the method’s efficacy in detecting dogs with CKD has not been established.

Based on our clinical experience, which includes many dogs with clinical and laboratory findings consistent with CKD but KL/AoD values within normal limits, we hypothesized that the KL/AoD ratio is a method with high specificity but low sensitivity. The primary aim of this study was to investigate the clinical usefulness of the KL/AoD method in detecting dogs with clinical and laboratory results consistent with CKD. The secondary aims were to verify if the previously reported cut-off values [7] were respected in our sample and to test the effects of variables such as side, body weight, and body size on the KL, AoD, and KL/AoD ratio in the healthy group.

## 2. Materials and Methods

### 2.1. Study Design

This study was prospective, single-institutional, analytical cross-sectional, and reference interval in design [10]. It received approval from the Clinical Ethical Review Board of the University of Napoli “Federico II” (PG/2023/PG0002892).

### 2.2. Ultrasonographic Examination Protocol

**Figure 1:**
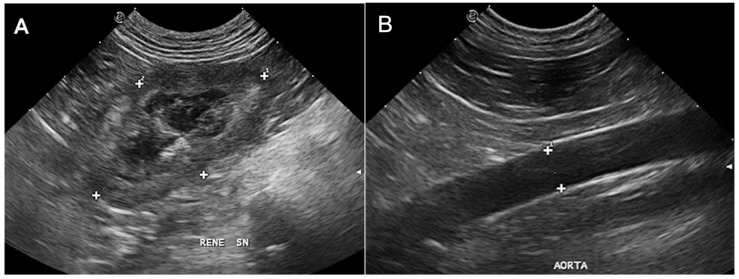
Dorsal ultrasonographic image of the left kidney (**A**) and of the aorta (**B**) acquired just caudal to the origin of the renal artery. In (**A**), the renal length is measured at the point of maximal cranio-caudal renal length (indicated by the measurement cursors with number 1). In (**B**), the maximal luminal diameter is obtained after reviewing cineloop frames to account for pulsation of the aorta; measurement cursors were placed at the margin of the lumen, excluding the vessel walls.

All the ultrasonographic (US) exams were performed at the Interdepartmental Center of Veterinary Radiology of the University of Napoli “Federico II” with the patient in dorsal or lateral recumbency, using the same ultrasound device (MyLab Class C Vet, Esaote, Genova, Italy). A microconvex or linear, multifrequency electronic probe (model SC3123, 3.5–10 MHz and model LA533, 3–13 MHz, respectively, Esaote, Genova, Italy) was used depending on the ultrasonographer’s preferences, which were mainly based on the best resolution achievable according to the patient’s size. All the US exams were performed by the same operator (L.M.), a professor of veterinary radiology with more than 26 years of experience in ultrasonography. The operator was unblinded regarding the patient’s clinical status and laboratory blood analysis results, if available during the exam. The KL/AoD ratio has been included in the institutional abdominal ultrasound examination protocol since May 2017 and has been assessed in all patients undergoing abdominal ultrasound using previously established methods [7]. Briefly, the KL was determined in the dorsal plane, from a subcostal or intercostal acoustic window, depending on patient conformation, and measured on a still frame acquired when the distance between the two poles was maximum and with the renal pelvis clearly delineated (Figure 1A). The aorta was assessed from the left flank, and images were acquired in a longitudinal scan just caudal to the root of the left renal artery. The measurements of the AoD were acquired at maximal luminal diameter, excluding the vessel walls, after reviewing the last frames using the cineloop function of the ultrasound device (Figure 1B).

### 2.3. Exclusion Criteria

Exclusion criteria were as follows: (a) US exam performed by a different operator; (b) measurements of both KL and AoD not obtained at the time of the US examination; (c) images deemed to be of inadequate quality (e.g., borders poorly defined, blurred contours); (d) the patient was less than twelve months of age; (e) serum renal function test (creatinine and urea) were not collected within 48 h before or after the US examination; (f) the patient was under sedation or general anesthesia; (g) the patient had ultrasonographic renal alterations consistent with congenital or acquired disease different from CKD (e.g., renal tumors, hydronephrosis, polycystic kidney disease, pyelonephritis, renal hematoma, renal abscess).

### 2.4. Data Recording

The images, signalment, history, and clinical data (including serum creatinine and urea levels) of patients undergoing US examination of the abdomen between May 2017 and May 2023 were retrieved from the picture archiving and communication system (dcm4chee-arc-light version 5.11.1, http://www.dcm4che.org) [11] and institutional electronic medical record.

The KL and AoD dimensions (in centimeters), breed, sex, weight (in kilograms), age (in years), and results of serum creatinine and urea levels (both expressed in mg/dL) were recorded for each dog included in the preliminary sample in an electronic spreadsheet (Microsoft Excel version 16.52 2021, Microsoft Corp., Redmond, WA, USA). The decision on whether to include or exclude patients from the final sample was made by one of the authors (D.C.), a veterinarian with a Ph.D. in Veterinary Sciences and 5 years of expertise in ultrasonography.

After being included in the final sample, the same author divided dogs into two main groups: “healthy” and “diseased”. The subjects included in the diseased group were required to have at least two consecutive tests with creatinine (threshold value of 1.4 mg/dL) and urea (threshold value of 60 mg/dL) levels above the reference values, with the last determination obtained within 48 h before or after the ultrasound examination [12]. Furthermore, dogs from the healthy group were subdivided into four sub-groups according to their body weight: toy (≤5.4 kg), small (5.5–10 kg), medium (10–25.9 kg), and large (≥26 kg).

### 2.5. Statistical Analysis

Statistical analyses were performed by one of the authors (D.C.), using commercial statistics software (Prism version 9.5.0 (525), GraphPad Software, San Diego, CA, USA and MedCalc version 19.2.6, MedCalc Software Ltd., Ostend, Belgium).

The D’Agostino–Pearson test was used to assess data for normality, and the data were further analyzed according to their distribution. Descriptive statistics, including the mean or median, range (minimum to maximum), standard deviation (SD), and 95% confidence interval (C.I.) of the mean, were calculated for age, body weight, right kidney length (RKL), left kidney length (LKL), AoD, RKL/AoD ratio, and LKL/AoD ratio.

In the healthy group, differences between the RKL and LKL were evaluated using the paired *t*-test and differences between the RKL/AoD and the LKL/AoD using the Wilcoxon signed-rank test. In the same group, differences in the KL and AoD among the toy, small, medium, and large dogs were assessed using one-way analysis of variance (ANOVA) with Welch’s correction, as variance results were significantly different at the F-test and further analyzed using the Dunnett’s multiple comparisons post hoc test. Differences in the KL/AoD ratio were assessed using the Kruskal–Wallis test, and the results were further analyzed using Dunn’s multiple comparison post hoc test. Furthermore, in the healthy group, the correlation between the body weight and KL, AoD, and the KL/AoD ratio was investigated using Spearman’s rank correlation coefficient (r_s_) and simple linear regression.

Finally, in the healthy group, the reference range for the KL/AoD ratio was calculated according to the American Society for Veterinary Clinical Pathology guidelines for reference intervals [13]. Outliers were automatically identified according to Reed et al., and then, data distribution was tested automatically with the D’Agostino–Pearson test [14]. Reference lower and upper limits, corresponding to the 2.5th and 97.5th fractiles and the corresponding 90% C.I., were then calculated employing nonparametric methods following Clinical Laboratory and Standards Institute recommendations (CLSI C28-A3) [15,16].

Differences between healthy and diseased groups in the KL and KL/AoD ratio were assessed using the Mann–Whitney U test. Finally, the diagnostic performance of the KL/AoD ratio to discriminate between healthy and diseased dogs was evaluated by receiver operating characteristic (ROC) curves, following the method proposed by DeLong et al., to obtain the associated area under the curve (AUC) and Youden index (J = sensitivity + specificity − 1). A J value < 0.2 indicates poor diagnostic performance, values between 0.2 and 0.5 indicate moderate diagnostic performance, and values > 0.5 indicate good diagnostic performance [17,18,19,20,21]. In all analyses, *p* < 0.05 was considered statistically significant.

## 3. Results

Of the 1703 US exams performed in the considered period, 227 dogs met the inclusion criteria (87 males, 19 castrated males, 62 females, 59 neutered females). In total, 185 (81.5%) (72 males, 16 castrated males, 54 female, 43 spayed females) were rated as healthy, and 42 (18.5%) (15 males, 3 castrated males, 8 female, 16 spayed females) as diseased.

In the healthy group, the mean age was 8 (±3) years, and the median body weight was 14 kg (range 1.8–65 kg). In the diseased group, the mean age was 9 (±4) years, and the median body weight was 18 kg (range 3–35 kg). The absolute number of dogs and the number of diseased dogs for each breed included in the final sample are summarized in Table 1. Descriptive statistics for the RKL, LKL, AoD, RKL/AoD, and LKL/AoD for both the healthy and diseased groups are summarized in Table 1.

In the healthy group, the paired *t*-test revealed no statistical differences between the RKL and LKL (*p* = 0.29). Similarly, the Wilcoxon signed-rank test showed no statistical differences between the RKL/AoD and LKL/AoD ratios (*p* = 0.05). Therefore, in the subsequent statistical analyses, the KL and KL/AoD ratios of the left and right sides were pooled together.

In the healthy group, based on their body weight, 30 (16.22%) were classified as toy-sized, 46 (24.86%) as small-sized, 69 (37.3%) as medium-sized, and 40 (21.62%) as large-sized. Descriptive statistics for the KL, AoD, and KL/AoD for each sub-group are summarized in Table 2.

**Figure 2:**
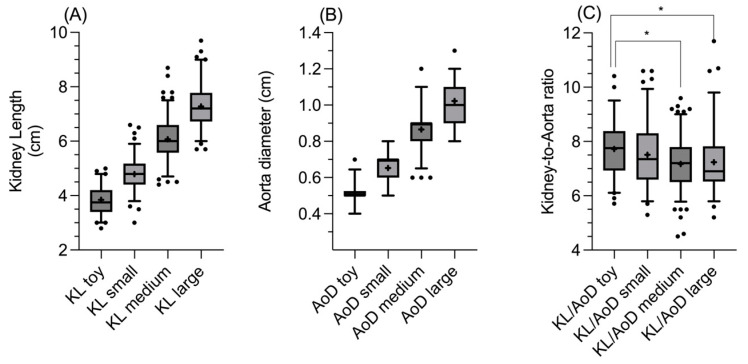
Box and whisker plot of one-way ANOVA of (**A**) KL (in cm), (**B**) AoD (in cm), and (**C**) Kruskal–Wallis test of KL/AoD ratio, for toy-, small-, medium-, and large-sized sub-groups. The line within the box represents the median, while the cross the mean, upper and lower sides of the box are the lower and upper quartiles, and the two extreme horizontal lines represent 5–95 percentiles. The asterisks (*) in Figure (**C**) indicate the statistically significant differences.

Furthermore, in the healthy group, the one-way ANOVA and Dunnett’s multiple comparison tests revealed statistically significant differences among all the four different sub-groups considered for KL (all *p* < 0.0001; Figure 2A) and AoD (all *p* < 0.0001; Figure 2B). Similarly, the Kruskal–Wallis and Dunn multiple comparison tests revealed a statistically significant difference in the KL/AoD ratio between the toy and medium groups and between the toy and large groups (*p* = 0.01 and *p* = 0.009, respectively; Figure 2C).

**Figure 3:**
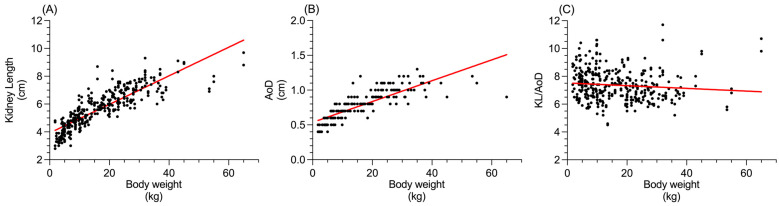
Correlation between body weight (in kg) and (**A**) KL (in cm), (**B**) AoD (in cm), and (**C**) KL/AoD ratio. The red solid line represents the simple linear regression.

The Spearman’s rank correlation coefficient revealed a positive correlation between the body weight and KL (*p* < 0.0001; r_s_ = 0.89; Figure 3A) and AoD (*p* < 0.0001; r_s_ = 0.90; Figure 3B), while a slight negative correlation was found between the body weight and KL/AoD (*p* = 0.0002; r_s_ = −0.19; Figure 3C).

The reference intervals, median, and range (minimum to maximum) with the relative 90% C.I. for the KL/AoD ratio in the healthy group are summarized in Table 3.

**Figure 4:**
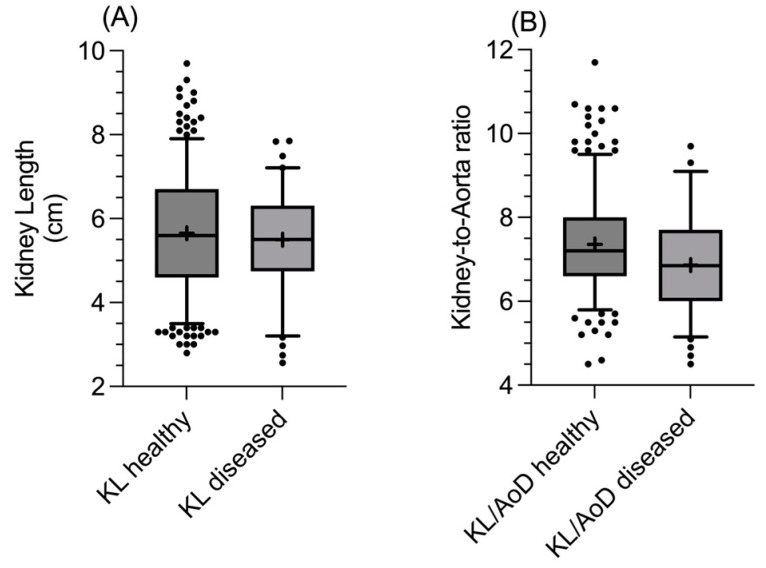
Box and whisker plot comparing (**A**) **the** kidney length (in cm) and (**B**) the KL/AoD ratio in healthy and diseased dogs. The line within the box represents the median, while the cross the mean, upper and lower sides of the box are the lower and upper quartiles, and the two extreme horizontal lines represent 5–95 percentiles.

The unpaired *t*-test did not reveal significant differences in the KL (*p* = 0.53; Figure 4A) and AoD (*p* = 0.44) between healthy and diseased dogs, while the Mann–Whitney U test found significant differences between the two groups for the KL/AoD ratio (*p* = 0.0003; Figure 4B), with diseased dogs having overall lower values.

**Figure 5:**
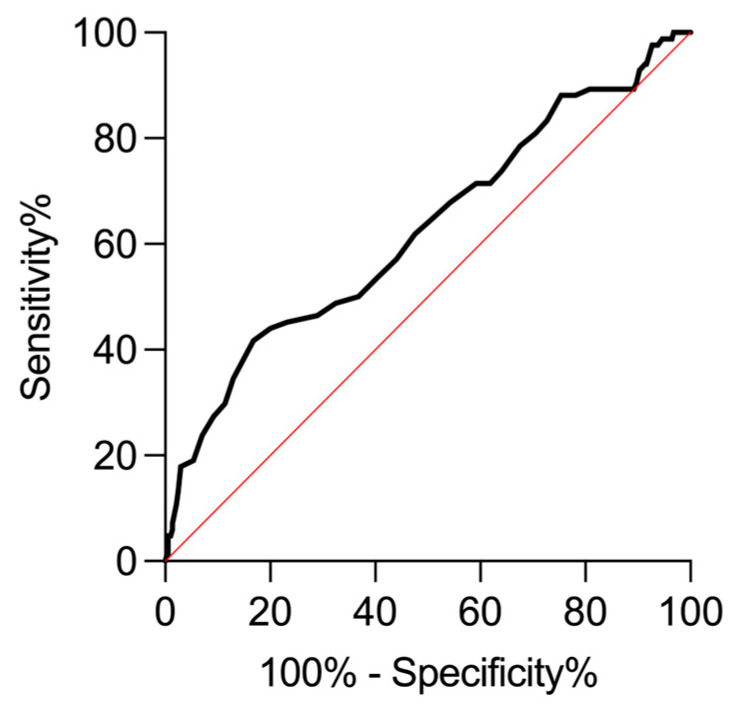
Receiver operating characteristic (ROC) curve of KL/AoD ratio.

The generated ROC curve for the KL/AoD ratio (Figure 5) displayed a fair AUC (AUC = 0.62; 95% C.I.: 0.55–0.69; *p* = 0.0003) and a moderate diagnostic performance (J = 0.25), considering the Youden index at KL/AoD = 6.3, where the ROC curve showed a specificity of 83.24% (95% C.I.: 79.10–86.70%) and a sensitivity of 41.67% (95% C.I.: 31.71–52.35%). For the lower value of the obtained reference range (KL/AoD = 5.6), the specificity increased to 97.57% (95% C.I.: 95.44–98.72%), while the sensitivity decreased to 13.10% (95% C.I.: 7.47–21.95%).

## 4. Discussion

This study evaluated the clinical usefulness of the KL/AoD ratio. It assessed the method’s sensitivity and specificity by comparing the results obtained from healthy dogs with those from clinical and laboratory results consistent with CKD. The study’s results supported our hypothesis.

The ROC curve demonstrated that for a KL/AoD ratio = 5.6 (i.e., the obtained lower reference limit), the specificity is very high (97.57%), while on the other hand, the sensitivity is very low (13.10%). The diagnostic performance of the method was barely moderate considering the Youden index (which combines sensitivity and specificity into a single measure of diagnostic test effectiveness) at a KL/AoD ratio = 6.3, where specificity remained excellent (83.24%), while the sensitivity increased considerably (41.67%).

As expected, the comparison of the KL between healthy and diseased dogs, without considering other variables, showed no significant differences, with overlapping values between the two groups. On the other hand, for the KL/AoD ratio, a statistically significant difference was found between healthy and diseased dogs, with the latter showing a lower median and 95% C.I. These results support using this method to normalize the renal dimensions among dogs of different sizes while also suggesting that dogs with CKD generally tend to exhibit a lower KL/AoD ratio. However, the KL/AoD ratio values obtained from the healthy and diseased groups overlap to a certain extent, limiting the clinical usefulness of the method in a clinical scenario. Indeed, dogs with initial CKD may not exhibit significant alterations in renal linear dimensions, causing partial overlap in the KL/AoD ratio values between the two groups and limiting the method sensitivity [22]. The obtained sensitivity and specificity values result in low false positives, making it highly likely that subjects with values below the reference range are genuinely affected by CKD. Therefore, the authors retain that the KL/AoD ratio is a valuable tool for confirming the diagnosis of CKD when clinical, laboratory, and additional ultrasonographic findings are consistent with the condition, allowing for greater diagnostic confidence while excluding other causes of elevated serum urea and creatinine levels, since these increased values are not exclusively indicative of CKD [23].

From our results, and as expected, the KL/AoD ratio is not suitable as a standalone screening method for CKD due to the partial overlap of values between healthy and diseased subjects, which leads to low sensitivity, and many diseased subjects may present values within the range of healthy individuals (false negatives). The reference ranges in our sample for the KL/AoD ratio (i.e., 5.6–9.9; Table 3) are broad and similar to those previously described (i.e., 5.5–9.2) [7]. This wide range is probably related to the randomness of the sample, as it is proved that dogs of the same breed tend to have narrower reference intervals [9].

To ensure the most accurate measurements, only images in which the cranial and caudal profiles of each kidney were clearly visible were included in the sample, and all measurements were taken during the examination rather than retrospectively. Although, in deep-narrowed chest dogs, the cranial profiles of the RK can be more difficult to delineate due to its subcostal position [2], in the current study, similar to previous studies [7,8,9] and differently from others [4,24,25], there were no differences between the RKL and LKL. Consequently, a single reference interval can be used for both kidneys.

In the original study by Mareschal et al., the AoD measurements acquired on longitudinal scans were more accurate than those acquired on transversal scans, showing a higher degree of inter-observer agreement [7]. On the contrary, in a later study on growing puppies, the authors suggested acquiring the AoD on transverse scans since, according to them, those are less affected by motion artefacts [25]. In a study that established the KL/AoD ratio reference interval in clinically healthy whippets, the authors did not find a significant difference between the two scanning planes [9]. In the present study, the AoD was acquired on longitudinal scans since, according to the literature, transversal scans can be affected by refraction artefacts that can alter image quality [26,27]. Although the method proved to have a good inter-operator agreement in the original study [7], we decided to include only the measurements performed by a single observer to limit the influence of this variable. Furthermore, we also decided to exclude from the final sample dogs that underwent US examination under sedation or general anesthesia, as the different sedation/anesthesia adopted protocols could affect blood pressure and, possibly, the AoD. Additionally, only adult dogs were included in the final sample because, as previously demonstrated, growing dogs, particularly those younger than six months, tend to have a higher KL/AoD ratio [25], and consequently alter the reference values.

The further subdivision of dogs by body size (toy, small, medium, large) revealed significant differences between the groups in KL and AoD. As expected, large dogs exhibited the largest dimensions for both KL and AoD. Interestingly, toy breeds showed statistically higher mean KL/AoD ratio values than medium and large breeds. This finding aligns with previous studies suggesting that renal dimensions tend to increase progressively with body size, though the rate of increase diminishes at the extremes of body weight, especially compared to aortic dimensions [4,28]. Consistently, in the present study, a positive correlation between body weight and KL was observed, though it became less pronounced at the higher extremes of body weight. These observations are further supported by the negative correlation between body weight and the KL/AoD ratio, confirming that at higher body weights, the AoD increases proportionally more than the KL.

The main limitations of our study include the lack of histopathological confirmation of the underlying cause of CKD affecting the dogs in the diseased group since this procedure is rarely performed at our institution. Although unlikely, it is possible that some dogs with altered serum urea and creatinine levels for causes unrelated to CKD were erroneously included in the diseased group.

A second limitation is the reduced number of dogs in the diseased group, resulting from our exclusion criteria where only dogs with a US study performed by the same operator and with laboratory analysis performed within 48 h from the US study were included. This low number of subjects included in the final sample and the lack of additional data necessary for the staging of the CKD, such as symmetric dimethylarginine levels, urine protein to creatinine ratio, and blood pressure, also prevented the staging of the dogs with CKD according to the IRIS guidelines [12] and the subdivision of the diseased dogs in sub-groups as for the healthy ones. Indeed, it would be interesting to assess whether the KL/AoD ratio tends to decrease progressively in subjects with advanced stages of CKD and if the subdivision among sub-groups could help increase the sensitivity of the method. Another limitation was the lack of information regarding the dogs’ hydration status and the presence of cardio-circulatory diseases that may alter the aorta distensibility and, thus, the KL/AoD ratio.

## 5. Conclusions

This study showed reference ranges for the KL/AoD ratio similar to those previously described for a sample of mixed subjects. This range is broad and limits the clinical usefulness of the method, suggesting the determination and use of breed-specific ratios that may be narrower. In the general canine population, a KL/AoD ratio = 6.3, although higher than the lower cut-off value, has good specificity and acceptable sensitivity in confirming CKD in dogs with clinical, laboratory, and additional US findings consistent with the disease. It must be kept in mind that the assessment of the renal length and renal linear dimensions, in general, is only one of the parameters to be considered during the US exam of the kidneys, and other alterations affecting the renal echotexture, echogenicity, and perfusion must also be taken into account [1,2,3,22]. Further studies involving a larger cohort of subjects with confirmed CKD are needed to evaluate variations in the KL/AoD ratio. These studies should consider additional factors such as breed, systemic blood pressure, inter-observer variability, different stages of CKD, operator experience, and the effects of systemic blood pressure and hydration on the measurements.

## Figures and Tables

**Table 1:** Descriptive statistics for right and left kidney length, aortic luminal diameter, and kidney-to-aorta ratios in the healthy and diseased groups.

	Healthy Group (*n* = 185)					Diseased Group (*n* = 42)				
	RKL (cm)	LKL (cm)	AoD * (cm)	RKL/AoD *	LKL/AoD	RKL (cm)	LKL (cm)	AoD (cm)	RKL/AoD	LKL/AoD
Mean (±SD)	5.7 ± 1.4	5.6 ± 1.4	0.79 ± 0.21	7.5 ± 1.1	7.2 ± 1.1	5.5 ± 1.2	5.4 ± 1.2	0.82 ± 0.19	6.9 ± 1.2	6.8 ± 1.1
Median	5.6	5.6	0.8	7.3	7.1	5.7	5.4	0.85	6.8	6.9
95% C.I.	5.5–5.9	5.4–5.8	0.76–0.82	7.3–7.6	7.1–7.4	5.2–5.9	5.1–5.8	0.76–0.87	6.6–7.3	6.5–7.2
Range (min–max)	3–9.7	2.8–9.1	0.4–1.3	4.6–12	4.5–11	2.6–7.9	2.7–7.8	0.47–1.1	4.5–9.3	4.7–9.7

Values indicated with asterisk (*) are non-normally distributed. Abbreviations: AoD, aortic luminal diameter; C.I. = confidence interval; cm = centimeters; LKL = left kidney length; LKL/AoD = left kidney-to-aorta ratio; max = maximum; min = minimum; RKL = right kidney length; RKL/AoD = right kidney-to-aorta ratio; SD = standard deviation.

**Table 2:** Descriptive statistics for kidney length, aortic luminal diameter, and kidney-to-aorta ratio (pooled data) for the healthy group’s four size sub-groups (toy, small, medium, and large).

Size Sub-Group		Mean (±SD)	Median	95% CI	Range (min–max)
Toy size (*n* = 30)	KL (cm) (*n* = 60)	3.8 ± 0.54	3.8	3.7–4	2.8–5
	AoD (cm) (*n* = 30)	0.51 ± 0.07	0.5	0.48–0.53	0.4–0.7
	KL/AoD (*n* = 60)	7.7 ± 1.1	7.8	7.4–8	5.7–10
Small size (*n* = 46)	KL (cm) (*n* = 92)	4.8 ± 0.62	4.8	4.7–4.9	3–6.6
	AoD (cm) (*n* = 46)	0.65 ± 0.08	0.7	0.63–0.68	0.5–0.8
	KL/AoD * (*n* = 92)	7.5 ± 1.2	7.4	7.3–7.8	5.3–11
Medium size (*n* = 69)	KL (cm) (*n* = 138)	6.1 ± 0.82	6	5.9–6.2	4.4–8.7
	AoD (cm) (*n* = 69)	0.87 ± 0.13	0.9	0.83–0.90	0.60–1.2
	KL/AoD (*n* = 138)	7.2 ± 0.95	7.2	7–7.3	4.5–9.6
Large size (*n* = 40)	KL (cm) (*n* = 80)	7.3 ± 0.85	7.2	7.1–7.5	5.7–9.7
	AoD (cm) (*n* = 40)	1 ± 0.13	1.0	0.98–1.1	0.80–1.3
	KL/AoD * (*n* = 80)	7.2 ± 1.2	6.9	7–7.5	5.2–12

Values indicated with asterisk (*) are non-normally distributed. Abbreviations: AoD, aortic luminal diameter; C.I. = confidence interval; cm = centimeters; KL = kidney length; KL/AoD = kidney-to-aorta ratio; max = maximum; min = minimum; SD = standard deviation.

**Table 3:** Median, upper, and lower limits of the reference value (and corresponding 90% C.I.) of the KL/AoD ratio in the healthy group.

	Median	Lower Limit (90% C.I.)	Upper Limit (90% C.I.)
KL/AoD	7.2	5.6 (5.3–5.8)	9.94 (9.6–10.6)

Values are non-normally distributed. Abbreviations: C.I. = confidence interval; KL/AoD = kidney-to-aorta ratio.

## Data Availability

Anonymized data supporting reported results are available from the corresponding author upon reasonable request.

## Abbreviations

ANOVA, analysis of variance; AoD, aortic luminal diameter; AUC area under the curve, C.I., confidence interval; CKD, chronic kidney disease; KL, kidney length; LKL, left kidney length; RKL, right kidney length; ROC receiver operating characteristic; SD, standard deviation; US, ultrasonographic.
